# Parity and endometrial cancer risk: a meta-analysis of epidemiological studies

**DOI:** 10.1038/srep14243

**Published:** 2015-09-16

**Authors:** Qi-Jun Wu, Yuan-Yuan Li, Chao Tu, Jingjing Zhu, Ke-Qing Qian, Tong-Bao Feng, Changwei Li, Lang Wu, Xiao-Xin Ma

**Affiliations:** 1 Department of Clinical Epidemiology, Shengjing Hospital of China Medical University, Shenyang 110004, Liaoning, China; 2 Department of Hematology, the Affiliated Hospital of Xuzhou Medical College, Xuzhou, Jiangsu, 221000, China; 3 Oncology Institute, the Affiliated Hospital of Nanjing Medical University, Changzhou No.2 People’s Hospital, Changzhou, Jiangsu, 213003, China; 4 Program of Quantitative Methods in Education, University of Minnesota, Minneapolis, Minnesota, 55455, USA; 5 Division of Epidemiology, Department of Medicine, Vanderbilt Epidemiology Center, Vanderbilt University School of Medicine, Nashville, TN 37203, USA; 6 Department of Epidemiology, Tulane University School of Public Health and Tropical Medicine, New Orleans, Louisiana, 70112, USA; 7 Center for Clinical and Translational Science, Mayo Clinic, Rochester, Minnesota, 55905, USA; 8 Department of Obstetrics and Gynecology, Shengjing Hospital of China Medical University, Shenyang 110004, Liaoning, China

## Abstract

The association between parity and endometrial cancer risk is inconsistent from observational studies. We aimed to quantitatively assess the relationship by summarizing all relevant epidemiological studies. PubMed (MEDLINE), Embase and Scopus were searched up to February 2015 for eligible case–control studies and prospective studies. Random-effects model was used to pool risk estimations. Ten prospective studies, 35 case-control studies and 1 pooled analysis of 10 cohort and 14 case-control studies including 69681 patients were identified. Pooled analysis revealed that there was a significant inverse association between parity and risk of endometrial cancer (relative risk (RR) for parous versus nulliparous: 0.69, 95% confidence interval (CI) 0.65–0.74; I^2^ = 76.9%). By evaluating the number of parity, we identified that parity number of 1, 2 or 3 versus nulliparous demonstrated significant negative association (RR = 0.73, 95% CI 0.64–0.84, I^2^ = 88.3%; RR = 0.62, 95% CI 0.53–0.74, I^2^ = 92.1%; and RR = 0.68, 95% CI 0.65–0.70, I^2^ = 20.0% respectively). The dose-response analysis suggested a nonlinear relationship between the number of parity and endometrial cancer risk. The RR decreased when the number of parity increased. This meta-analysis suggests that parity may be associated with a decreased risk of endometrial cancer. Further studies are warranted to replicate our findings.

As the most common tumor of the female reproductive tract, endometrial cancer remains the fourth most common malignancy in females^1^. Parity, a representative reproductive factor, is demonstrated to potentially modulate risk of endometrial cancer through affecting estrogen and progesterone levels^2^. A lot of observational studies also suggest such an association. For example, in comparison to nulliparous, parous was detected to be associated with decreased risk of developing endometrial cancer in several prospective studies^3^,^4^, case-control studies^5^,^6^,^7^,^8^,^9^,^10^,^11^,^12^, as well as pooled analysis^13^. However, such an inverse association was not detected in several other epidemiological studies^14^,^15^,^16^,^17^.

Considering that results from individual epidemiological studies can be strongly affected by available sample sizes, a better way to clarify the association between parity and risk of endometrial cancer is to summarize all available evidence from relevant observational studies. In the current study, we aimed to conduct a comprehensive meta-analysis to evaluate this research question. We also conducted analysis to clarify the dose-response relationship between number of parity and risk of endometrial cancer.

## Results

### Literature Search and Study Characteristics

**Figure 1:**
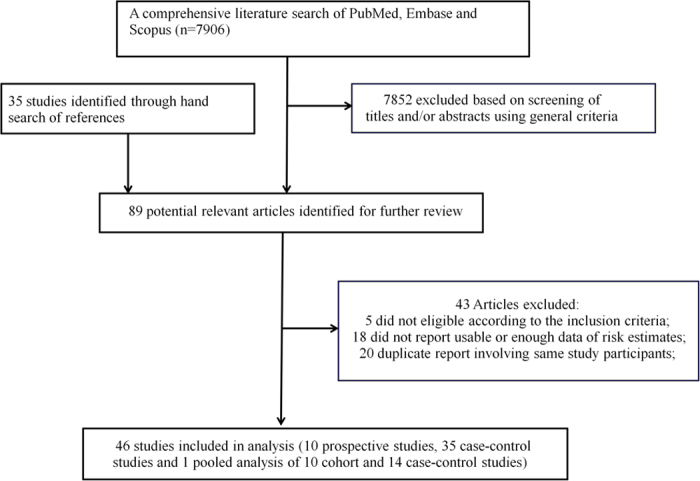
Flow chart for selection of eligible studies.

The detailed procedures of the literature search and article screening were demonstrated in Fig. 1. The database search yielded 7906 publications, among which 7852 were excluded based on the screening of titles and/or abstracts. Combined with 35 studies identified through manual search of references of relevant review articles, the whole contents of a total of 89 publications were assessed. Among them, 43 articles were further excluded due to various reasons: 5 did not meet the eligibility criteria; 20 involved duplicated study individuals with other articles; and 18 did not report sufficient data or information (the complete list of the 43 excluded articles is available upon request). Finally, a total of 46 studies were included in the current meta-analysis (references are within the supplementary material). The detailed characteristics of the involved studies were demonstrated in Table 1. In total, 10 prospective cohort studies, 35 case-control studies and 1 pooled analysis of 10 cohort and 14 case-control studies were involved. Overall, 18 studies were conducted in Europe, 18 in America, 9 in Asia, and 1 was an international report. The studies enrolled 69681 patients. The quality assessments of these studies were demonstrated in Tables 2 and 3. Overall, 9 of the 10 cohort studies (90%) and 26 of the 35 case-control studies (74%) were categorized as high-quality studies. Others were categorized as low-quality studies.

### Parous vs. Nulliparous

**Figure 2:**
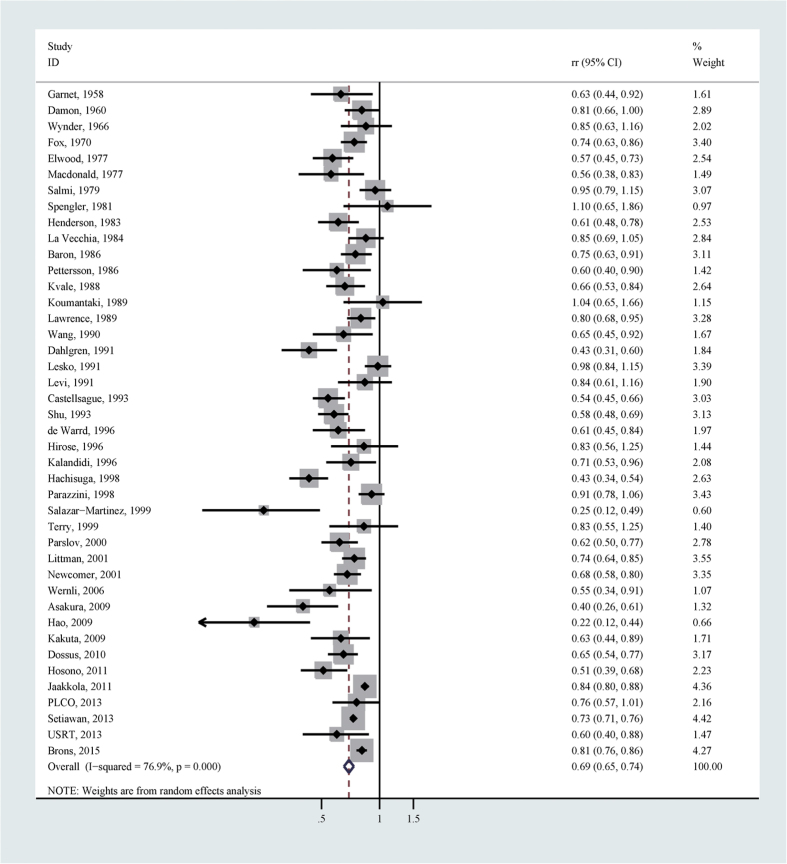
Forest plot (random effects model) of parity (parous vs. nulliparous) and endometrial cancer risk.

A total of 42 studies reported the association between risk of endometrial cancer and parity for parous versus nulliparous. After summarizing all available estimates, there was a significant inverse association between parity and endometrial cancer risk (relative risk (RR)  = 0.69, 95% confidence interval (CI) 0.65–0.74), with considerable heterogeneity (I^2^ = 76.9%; Table 4 **and** Fig. 2). There was no significant publication bias as suggested by Begg’s test (*p* for bias: 0.104). Sensitivity analysis revealed that the 42 study-specific RRs of parous versus nulliparous ranged from as low as 0.69 (95% CI 0.64–0.73; I^2^ = 76.9%) after omitting the study by Setiawan *et al.*^13^ to as high as 0.70 (95% CI 0.67–0.75; I^2^ = 74.2%) after omitting the study by Hachisuga *et al.*^11^. The subgroup analyses revealed that the significant negative association was detected in all strata according to study design, location, number of cases, study publication time, estimate adjustment, control resources and study quality (Table 4), although in a lot of subgroups the high heterogeneity persisted. According to the Galbraith plot (Supplementary Figure 1), 14 studies contributed to the heterogeneity^7^,^9^,^10^,^11^,^15^,^18^,^19^,^20^,^21^,^22^,^23^,^24^,^25^,^26^. After excluding these studies from the pooled analysis, the overall effect size remained similar (RR = 0.73, 95% CI 0.71–0.75), with no heterogeneity (I^2^ = 0.0%).

### Different number of parity

The associations between different number of parity (1, 2 or 3) and endometrial cancer risk were evaluated respectively. Parity number of 1 versus nulliparous was inversely associated with risk of endometrial cancer (RR = 0.73, 95% CI 0.64–0.84; I^2^ = 88.3%), after summarizing estimates from 19 studies (Table 5). The significant inverse association was detected in almost all strata of subgroup analyses (Table 5). According to the Galbraith plot (Supplementary Figure 2), 6 studies contributed to the heterogeneity^10^,^13^,^26^,^27^,^28^,^29^. The heterogeneity disappeared after excluding these studies in the pooled analysis (I^2^ = 0.0%). Similarly, after summarizing 13 studies, parity number of 2 versus nulliparous demonstrated a significant inverse association with risk of endometrial cancer (RR = 0.62, 95% CI 0.53–0.74; I^2^ = 92.1%), which was also identified in different strata of subgroup analyses (Table 6). Five studies contributed to the heterogeneity according to the Galbraith plot (Supplementary Figure 3)^6^,^10^,^13^,^26^,^29^. The heterogeneity disappeared after excluding these studies in the pooled analysis (I^2^ = 0.0%). Additionally, parity number of 3 versus nulliparous showed a significant inverse association with endometrial cancer risk (RR = 0.68, 95% CI 0.65–0.70; I^2^ = 20.0%), after pooling 7 studies.

### Dose-response analysis

**Figure 3:**
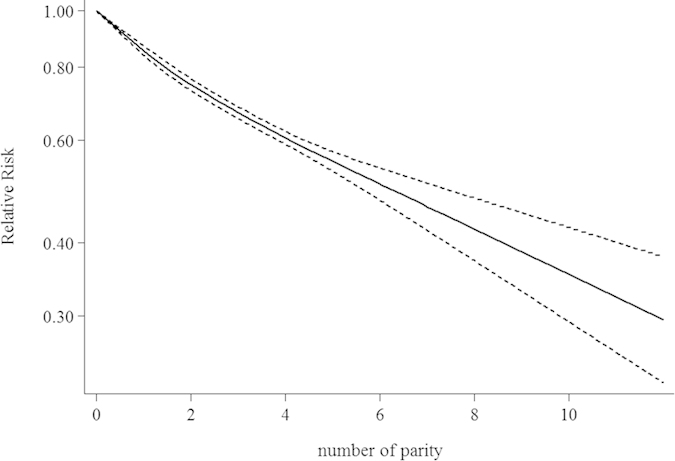
Nonlinear dose-response relationship between number of parity and endometrial cancer risk. The solid line represents the estimated relationship. The dashed line represents the 95% confidence interval of the estimated relationship.

Assuming a linear relationship, we detected that the combined RR per an additional live birth was 0.86 (95% CI 0.84–0.89), with considerable heterogeneity (P for heterogeneity < 0.0001). After testing a potential non-linear relationship, the test for nonlinearity suggested that a non-linear relationship might exist (p for nonlinearity: 0.0058). Under this model the RR also decreased when the number of parity increased. The nonlinear relationship between the number of parity and endometrial cancer risk in females was demonstrated in Fig. 3.

## Discussion

We performed a comprehensive quantitative meta-analysis to evaluate the relationship between parity and endometrial cancer risk. After summarizing all available evidence, ever giving birth to children was associated with an inverse risk of developing endometrial cancer. The sensitivity analysis demonstrated that the result was not significantly affected by any individual study; also subgroup analyses revealed that the inverse association was detected in all strata. Additionally, analyses assessing each number of parity (1, 2 and 3) demonstrated that the inverse association persisted for all 3 scenarios. Furthermore, we identified a dose-response relationship between the number of parity and risk of endometrial cancer. Overall, our findings support that parity may be associated with risk of endometrial cancer.

Our findings are plausible based on understandings from basic research. Estrogens are known to stimulate proliferation of cells in the endometrium and increase mitotic activity, which can induce cancer development^30^,^31^. On the other hand, progestins can decrease risk of developing endometrial cancer through reducing cell proliferation and stimulating differentiation^31^. During live birth, there is a hormonal balance shift toward less estrogen and more progesterone, which may further affect risk of developing endometrial cancer^32^. Our finding of the dose-response relationship between the number of parity and endometrial cancer risk may be attributable to repeatedly long-term progesterone actions for the antiestrogenic endometrial effects^33^,^34^. Another potential explanation is that at each birth delivery there is mechanical shedding of malignant/premalignant endometrial cells^28^,^35^.

Our study has several strengths. To the best of our knowledge, this is the first comprehensive meta-analysis evaluating the association between parity and endometrial cancer. Besides conducting subgroup analyses and sensitivity analysis to further evaluate the association, we assessed associations of different numbers of parity and conducted dose-response analysis to fully understand the relationship. Our analyses suggested that the finding of the inverse association between parity and endometrial cancer risk might be robust.

Several potential limitations need be acknowledged for the appropriate interpretation of our findings. First, we do not have access to the individualized primary data from each of the included studies, which induces the possibility that the risk estimates used in our pooled analysis may not be fully adjusted for. For example, obesity and use of oral contraceptive are among the known factors affecting risk of developing endometrial cancer^32^,^36^. However, in some of the included studies, they were not adjusted for the association between parity and endometrial cancer risk. Residual confounding may thus be an issue. Second, for the dose-response analysis, the highest categories of number of live birth have wide range of values in different studies. The exposure values may not be accurately assigned based on our assumptions in the methods section. However, this limitation is difficult to eliminate and the method we used is in concordance with the general approach in this area. Third, our study mainly summarizes evidence from observational studies, which are known to confer several relevant biases due to the observational nature. Further large scale multi-center prospective studies are warranted to replicate our findings. Forth, we notice considerable heterogeneities across studies in our pooled analyses. We conducted numerous subgroup analyses with the hope of detecting potential factors for such heterogeneities; however, it appears that in many subgroups the heterogeneity remains relatively high. According to the Galbraith plots, a proportion of the included studies contribute to the high heterogeneities. The heterogeneities disappear after excluding these studies in the pooled analyses. These need to be considered when interpreting our findings. Last, we would like to acknowledge that I^2^ value should be interpreted with caution because it has certain uncertainty. The value has relatively low statistical power especially in scenarios of small numbers of available studies^37^. However, in the current meta-analysis there are a relatively large number of eligible studies. Thus the possibility of this limitation is low.

In conclusion, based on a summarization of all available evidence from epidemiological studies, parous versus nulliparous was inversely associated with risk of endometrial cancer. There was a nonlinear dose-response relationship between the number of live births and risk of endometrial cancer. Our findings suggested that parity might be a risk factor for endometrial cancer, suggesting roles of reproductive factors in the etiology of endometrial cancer.

## Materials and Methods

### Data Sources and Search Strategies

A literature search of PubMed (MEDLINE), Embase and Scopus databases was conducted from the inception to February 2015. We used the following search keywords: (((((((((parity) OR pregnancy) OR livebirth) OR reproductive) OR reproduction) OR reproductive factors) OR reproductive factor)) AND ((endometrium) OR endometrial)) AND ((((((((((malignancies) OR malignancy) OR neoplasm) OR neoplasms) OR cancer) OR cancers) OR adenoma) OR adenomas) OR carcinoma) OR carcinomas). We also screened references of included articles and relevant review papers to identify other potential studies.

### Study Selection

Studies were eligible if they (i) were prospective studies or case–control studies or pooled analysis of epidemiological studies; (ii) evaluated the association between parity and risk of endometrial cancer; (iii) presented RR, odds ratio (OR), or hazard ratio (HR) values with 95% CI or necessary data for determination. Cross-sectional studies were excluded. Epidemiological studies comparing endometrial cancer cases with controls with gynecology conditions were excluded as well. If we identified multiple articles involving same participants, the study with the largest number of patients and most relevant information was included.

### Data Extraction and Quality Assessment

Two investigators independently carried out the abstract screening, full text screening, and data extraction. Disagreements were resolved by discussion, with input from other investigators. Data extracted from each study included: the first author’s name, publication year, study country, study design, characteristics of study population (sample size, age, length of follow-up, measures and numbers of parity, and association effect sizes). If more than 1 estimate were reported, we used the estimate that was adjusted for the most appropriate covariates, like the previous studies^38^,^39^,^40^,^41^,^42^. In situations where only unadjusted estimates were provided, we used the crude estimate in the analysis.

The qualities of included studies were assessed with the Newcastle-Ottawa Quality Assessment Scale^43^. Specifically, aspects of population and sample methods, exposure and outcome descriptions, and statistical matching/adjustments of the data were assessed. With this scale each study was assigned a score (maximum score is 9 points). Studies with an overall score of higher than or equal to 7 points were categorized as high-quality studies; others were categorized as low-quality studies.

### Statistical Methods

The RR and 95% CI from included studies were used as the measure of association. Due to the rarity of endometrial cancer, ORs and HRs were deemed equivalent to RRs and RRs were used to represent measures. I^2^ was used to assess the heterogeneity across studies, where a I^2^>50% suggests considerable heterogeneity^44^. We pooled the log transformed RR using the fixed-effects model^45^ when there was no considerable heterogeneity. We used the random-effects model^46^ when there was high heterogeneity. Besides pooling results for parous vs. nulliparous, we summarized effect sizes according to different numbers of parity. We evaluated parity number of 1 vs. nulliparous, parity number of 2 vs. nulliparous, and parity number of 3 vs. nulliparous respectively, according to the characteristics of the included studies. Subgroup analyses were conducted according to design of study (case-control vs. prospective studies), study location (America, Europe, Asia or International), number of cases (<200 vs. ≥200), study publication time (earlier than 1992 vs. 1992-), estimate adjustment, control source, and study quality (high-quality vs. low-quality) . We also conducted sensitivity analyses excluding one study at a time to explore whether any specific study strongly affected the results.

With regards to the dose-response analysis, we explored potential linear relationship between the number of parity and risk of endometrial cancer^47^. If studies reported the parity number by ranges, we used the midpoint of each category in the analysis. For studies in which the highest category did not have an upper end, the width of the highest category was assumed to be the same as the adjacent category, like previous studies^48^,^49^. Furthermore, we assessed potential non-linear relationship for the association. For this analysis, fractional polynomial models with restricted cubic splines and 3 knots at fixed percentiles (10%, 50%, and 90%) of the distribution were used^50^,^51^. We then performed a likelihood ratio test to determine whether nonlinear or linear relationship was suggested.

Publication bias was evaluated via Begg’s test^52^. A *P*-value of 0.05 was used as the threshold to determine significant publication bias. All statistical analyses were performed with Stata (version 13; StataCorp, College Station, TX).

## Additional Information

**How to cite this article**: Wu, Q.-J. *et al.* Parity and endometrial cancer risk: a meta-analysis of epidemiological studies. *Sci. Rep.* **5**, 14243; doi: 10.1038/srep14243 (2015).

## Supplementary Material

Supplementary Information

## Figures and Tables

**Table 1:** Characteristics of studies evaluating parity with endometrial cancer risk.

**First author’s last name, publication year, Country, Study design**		**Cases/subject (age), duration of follow up**	**Parity categories (exposure/case assessment)**	**RR (95% CI)**	**Matched/Adjusted factors**
**Prospective studies**					
Setiawan, 2013,International, 10 cohort and 14 case-control studies		14,069/35,312 (mean from 54.6–71.6y)	Nulliparous	1.0 (ref.)	unadjusted
			parous	0.73 (0.71–0.76)	
			1	0.88 (0.84–0.92)	
			2	0.78 (0.75–0.81)	
			3	0.68 (0.65–0.70)	
			≥4	0.60 (0.57–0.64)	
			(questionnaire or interview/cancer registry, pathology report, medical chart or slide review)		
Dossus, 2010, Europe, CS		1,017/302,618 (mean 50.5y), mean 8.7y	Nulliparous	1.0 (ref.)	Age, study center, body mass index (BMI), physical activity, alcohol, diabetes, smoking status and education
			Parous	0.65 (0.54–0.77)	
			Parity = 1	1.0 (ref.)	
			2	0.92 (0.76–1.11)	
			3	0.80 (0.64–0.99)	
			≥4	0.58 (0.44–0.78)	
			(Self-questionnaire/Cancer registry, histology confirmation)		
Wernli, 2006, China, CS		206/267,400 (N/A), mean 7.6y	Nulliparous	3.95 (1.43–10.86)	Age at baseline
			1	1.00 (ref.)	
			2	0.77 (0.42–1.42)	
			3	1.07 (0.57–2.04)	
			4	0.93 (0.46–1.86)	
			≥5	0.75 (0.36–1.56)	
			(Trained interviewer/Cancer registry and medical record )		
Hinkula, 2002, Finland, CS		419/86,978 (N/A), mean 19.3y	Parity number		Age at first birth, birth intensity
			5	1.0 (ref.)	
			6	0.72 (0.57–0.92)	
			7	0.87 (0.62–1.22)	
			≥8	0.71 (0.57–1.02)	
			(Registry/Cancer registry)		
					physical activity, fruit and vegetable consumption, diabetes, social-economic status, cigarette smoking, alcohol consumption
Terry, 1999, Sweden, CS		133/11,659 (median56.2y), mean 20.4y	Nulliparous	1.0 (ref.)	
			parous	0.83 (0.55–1.25)	
			1–2	0.9 (0.6–1.5)	
			≥3	0.4 (0.2–0.8)	
			(Self-questionnaire/Cancer registry)		
Albrektsen, 1995, Norway, CS		554/765,756 (30–56y), mean 12.2y	Nulliparous	1.94 (1.46–2.59)	Age, birth cohort
			1	1.00	
			2	0.84 (0.64–1.09)	
			3	0.61 (0.46–0.82)	
			≥4	0.48 (0.34–0.69)	
			(Registry/cancer registry)		
Kvale, 1988, Norway, CS		420/62,079 (27–69y), 19y	Nulliparous	1.0 (ref.)	Age, urban/rural place of residence
			parous	0.66 (0.53–0.84)	
			1	0.80 (0.59–1.10)	
			2	0.72 (0.55–0.96)	
			3	0.55 (0.39–0.77)	
			4	0.72 (0.50–1.06)	
			≥5	0.41 (0.26–0.66)	
			(Trained interviewer/Cancer registry)		
PLCO, US, CS		417/40562 (mean 62.8y), ~13y	Nulliparous	1.0 (ref.)	birth year and entry year, age at last menstrual period, age at menarche, BMI, oral contraceptive use, menopausal hormone therapy use, diabetes, and smoking status
			parous	0.76 (0.57–1.01)	
			(questionnaire/cancer registry and questionnaire)		
USRT, US, CS		125/10050 (mean ~57y), ~15y	Nulliparous	1.0 (ref.)	birth year and entry year, age at last menstrual period, age at menarche, BMI, oral contraceptive use, menopausal hormone therapy use, diabetes, and smoking status
			parous	0.60 (0.40–0.88)	
			(questionnaire/database link and questionnaire)		
de Warrd, 1996, Netherlands, CS		147/1047 (40–65y), up to 18 y	Nulliparous	1.0 (ref.)	unadjusted
			Parous	0.61 (0.45–0.84)	
			1–2	0.74 (0.52–1.04)	
			≥3	0.49 (0.33–0.72)	
			(questionnaire/database link)		
Bevier, 2011, Sweden, CS		31118/5759120 (NA), up to 45 y	Nulliparous	1.0 (ref.)	age, period, region, socioeconomic status
			1	0.47 (0.42–0.52)	
			2	0.41 (0.37–0.46)	
			3–4	0.36 (0.32–0.40)	
			5–9	0.29 (0.25–0.34)	
			10+	0.25 (0.10–0.58)	
			(database/database link)		
**First author, publication year**, Country, Study design		**Cases/control** (**age)**	**Parity categories** (**exposure/case assessment)**	**RR** (**95% CI)**	**Matched/Adjusted factors**
Case-control studies									
Parslov, 2000, Denmark, PC-CS	Nulliparous	1.0 (ref.)	Age, residence, family history of endometrial cancer, BMI, diabetes mellitus, hypertension, menarche, pregnancy, number of pregnancy, number of induced abortions, age of first birth, hyperandrogenism, amenorrhea, oral contraceptive use, hormone replacement therapy, cigarette smoking, and years of schooling						
			parous	0.62 (0.50–0.77)					
			1	0.6 (0.3–1.1)					
			2	0.3 (0.2–0.6)					
			≥3	0.2 (0.1–0.4)					
			(Self-questionnaire/histology confirmation)						
Salazar-Martinez, 1999, Mexico, HC-CS	85/668 (54.9y)		Nulliparous	1.0 (ref.)	Age, hormonal use, breastfeeding, smoking, diabetes mellitus, hypertension, physical activity, menopausal status, BMI				
			parous	0.25 (0.12–0.49)					
			1–2	0.41 (0.19–0.86)					
			3–4	0.15 (0.06–0.36)					
			≥5	0.16 (0.06–0.40)					
			(Trained interviewer/biopsy confirmation)						
Parazzini, 1998 Italy, HC-CS	752/2,606 (25–74y)		Nulliparous	1.0 (ref.)	Age, calendar year at interview, education, BMI, menopausal status, use of hormonal replacement therapy, smoking, history of diabetes, hypertension, abortions, age at first birth, time since last birth				
			parous	0.91 (0.78–1.06)					
			1	0.9 (0.7–1.1)					
			2	0.8 (0.6–1.0)					
			≥3	0.7 (0.5–0.8)					
			(Trained interviewer/histology confirmation)						
Kalandidi, 1996, Greece, HC-CS	145/298 (NA)		Nulliparous	1.0 (ref.)	Age, schooling, occupation, age at menopause, age at menarche, oral contraceptive, menopausal estrogen, smoking, alcohol intake, coffee intake, BMI, energy intake				
			parous	0.71 (0.53–0.96)					
			1	0.75 (0.27–2.11)					
			2	0.66 (0.26–1.67)					
			3	0.36 (0.13–1.03)					
			≥4	0.34 (0.11–1.05)					
			(Trained interviewer/histologic confirmation)						
Shu, 1993, China, PC-CS	268/268 (18–74y)		Nulliparous	1.0 (ref.)	Age				
			parous	0.58 (0.48–0.69)					
			1	0.3 (0.1–0.8)					
			2–3	0.2 (0.1–0.7)					
			≥4	0.1 (0.1–0.4)					
			(Trained interviewer/Cancer registry)						
Koumantaki, 1989, Greece, HC-CS	83/164 (40–79y)		Nulliparous	1.0 (ref.)	unadjusted				
			parous	1.04 (0.65–1.66)					
			1–2	1.19 (0.73–1.94)					
			≥3	0.81 (0.47–1.43)					
			(Trained interviewer/Biopsy-confirmation)						
Kelsey, 1982, US, HC-CS	167/903 (45–74y)		Nulliparous	1.0 (ref.)	Race, education, age at menopause, weight, history of diabetes, oral contraceptive use, age, menopausal status, estrogen replacement therapy use				
			1	0.8 (0.7–0.9)					
			(Trained interviewer/pathology confirmation)						
Baron, 1986, US, HC-CS	476/2128 (40–89y)		Nulliparous	1.0 (ref.)	unadjusted				
			parous	0.75 (0.63–0.91)					
			1–2	0.85 (0.69–1.05)					
			3–4	0.68 (0.54–0.86)					
			≥5	0.70 (0.55–0.90)					
			(interview/clinic diagnosis)						
Castellsague, 1993, US, PC-CS	437/3200 (20–54y)		Nulliparous	1.0 (ref.)	Location, age, time interval				
			parous	0.54 (0.45–0.66)					
			1–2	0.59 (0.48–0.74)					
			3–4	0.54 (0.43–0.68)					
			≥5	0.41 (0.29–0.59)					
			(interview/histological confirmation)						
Dahlgren, 1991, Sweden, PC-CS	147/1409 (31–65y)		Nulliparous	1.0 (ref.)	unadjusted				
			parous	0.43 (0.31–0.60)					
			(interview and/or questioinnaire/hospital records)						
Damon, 1960, US, HC-CS	197/233 (NA)		Nulliparous	1.0 (ref.)	unadjusted				
			parous	0.81 (0.66–0.995)					
			(hospital records/pathology diagnosis)						
Elwood, 1977, US, PC-CS	212/1198 (40–89y)		Nulliparous	1.0 (ref.)	age				
			parous	0.57 (0.45–0.73)					
			1	0.74 (0.49–1.13)					
			2	0.61 (0.44–0.86)					
			3	0.51 (0.33–0.76)					
			4+	0.48 (0.33–0.70)					
			(Questionnaire/histological confirmation)						
Fox, 1970, US, PC-CS	300/300 (NA)		Nulliparous	1.0 (ref.)	age				
			parous	0.74 (0.63–0.86)					
			(records/histological confirmation)						
Garnet, 1958, US, HC-CS	50/50 (30–80y)		Nulliparous	1.0 (ref.)	unadjusted				
			Parous	0.63 (0.44–0.92)					
			1–3	0.56 (0.37–0.85)					
			4+	0.95 (0.59–1.51)					
			(unclear/clinic diagnosis)						
Henderson, 1983, US, PC-CS	110/110 (45y−)		Nulliparous	1.0 (ref.)	age				
			Parous	0.61 (0.48–0.78)					
			1	0.91 (0.66–1.24)					
			2	0.70 (0.52–0.95)					
			3	0.51 (0.34–0.79)					
			4+	0.33 (0.18–0.60)					
			(trained interviewer/microscopical confirmation)						
Hirose, 1996, Japan, HC-CS	145/26751 (20y+)		Nulliparous	1.0 (ref.)	Age, first-visit year				
			Parous	0.83 (0.56–1.25)					
			1	0.63 (0.35–1.14)					
			2	0.62 (0.40–0.96)					
			3+	0.41 (0.25–0.69)					
			(questionnaire/histology diagnosis)						
Hosono, 2011, Japan, HC-CS	222/2162 (mean 56y)		Nulliparous	1.0 (ref.)	Age, menstrual-status				
			Parous	0.51 (0.39–0.68)					
			1–2	0.56 (0.42–0.74)					
			≥3	0.40 (0.27–0.60)					
			(questionnaire/histological confirmation)						
Jaakkola, 2011, Finland, PC-CS	7261/19490 (50–80y)		Nulliparous	1.0 (ref.)	age				
			Parous	0.84 (0.80–0.88)					
			1–2	0.90 (0.85–0.94)					
			≥3	0.76 (0.72–0.80)					
			(registry/cancer registry)						
Kakuta, 2009, Japan, HC-CS	152/285 (mean ~54y)		Nulliparous	1.0 (ref.)	Age, area of residence				
			Parous	0.63 (0.44–0.89)					
			1–3	0.94 (0.65–1.36)					
			≥4	0.89 (0.55–1.44)					
			(questionnaire/histopathological confirmation)						
Lawrence, 1989, US, PC-CS	84/168 (40–69y)		Nulliparous	1.0 (ref.)	Age, county of residence, weight, time since last medical visit, education, diabetes, estrogen pill use				
			Parous	0.80 (0.68–0.95)					
			(Trained interviewer/medical record review)						
Lesko, 1991, US, HC-CS	483/693 (30–69y)		Nulliparous	1.0 (ref.)	Age, race, religion, BMI, diabetes history, hypertension history, alcohol use, tobacco use, durations of oral contraceptive and non-contraceptive estrogen use, menopausal status, age at menopause, age at first pregnancy, years of education, date of interview, geographic region				
			Parous	0.98 (0.84–1.15)					
			1–2	1.3 (0.9–1.9)					
			3–4	1.0 (0.7–1.5)					
			≥5	0.5 (0.3–0.9)					
			(Trained interviewer/clinic diagnosis)						
Levi, 1991, Switzerland, HC-CS	122/309 (75y−)		Nulliparous	1.0 (ref.)	unadjusted				
			Parous	0.84 (0.61–1.16)					
			(Trained interviewer/histological confirmation)						
Littman, 2001, US, PC-CS	679/944 (45–74y)		Nulliparous	1.0 (ref.)	Age, location				
			Parous	0.74 (0.64–0.85)					
			1	0.91 (0.75–1.11)					
			>1	0.71 (0.62–0.82)					
			(Trained interviewer//histological confirmation)						
Macdonald, 1977, US, PC-CS	145/580 (unknown)		Nulliparous	1.0 (ref.)	age				
			Parous	0.56 (0.38–0.83)					
			(Medical record linkage/pathology confirmation)						
Newcomer, 2001, US, PC-CS	740/2372 (40–79y)		Nulliparous	1.0 (ref.)	age				
			Parous	0.68 (0.58–0.80)					
			1–2	0.8 (0.6–1.0)					
			3–4	0.6 (0.5–0.8)					
			≥5	0.4 (0.3–0.6)					
			(Trained interviewer/registry link and histologic confirmation)						
Pettersson, 1986, Sweden, PC-CS	254/254 (30–94y)		Nulliparous	1.0 (ref.)	Age, county of residence				
			Parous	0.6 (0.4–0.9)					
			1	0.7 (0.4–1.2)					
			2	0.7 (0.4–1.1)					
			3	0.6 (0.3–1.1)					
			4	0.4 (0.2–0.8)					
			≥5	0.3 (0.1–0.6)					
			(Trained interviewer/histologic confirmation)						
Spengler, 1981, Canada, PC-CS	88/177 (40–74y)		Nulliparous	1.0 (ref.)	age				
			Parous	1.10 (0.65–1.86)					
			(Trained interviewer/pathology confirmation)						
Wynder, 1966, US, HC-CS	112/200 (unknown)		Nulliparous	1.0 (ref.)	unadjusted				
			Parous	0.85 (0.63–1.16)					
			1	1.09 (0.73–1.64)					
			2	0.65 (0.42–1.01)					
			3	0.86 (0.53–1.38)					
			4	0.96 (0.53–1.73)					
			5	1.51 (0.71–3.20)					
			6	1.88 (1.02–3.48)					
			7	0.31 (0.05–1.99)					
			(Trained interviewer/histologic diagnosis)						
Wang, 1990, China, HC-CS	102/102 (mean 58y)		Nulliparous	1.0 (ref.)	Same hospital, time at diagnosis, age, marriage status				
			Parous	0.65 (0.45–0.92)					
			1–2	0.81 (0.55–1.20)					
			3–4	0.59 (0.39–0.88)					
			≥5	0.58 (0.38–0.91)					
			(Trained interviewer/pathology confirmation)						
Hachisuga, 1998, Japan, HC-CS	242/1021 (20–79y)		Nulliparous	1.0 (ref.)	Age, BMI, hypertension, diabetes				
			Parous	0.43 (0.34–0.54)					
			1–3	0.23 ((0.16–0.34)					
			≥4	0.33 (0.23–0.48)					
			(Medical record/histology comfirmation)						
Brons, 2015, Denmark, PC-CS	5382/72127 (30–84y)		Nulliparous	1.0 (ref.)	Age				
			Parous	0.81 (0.76–0.86)					
			1	0.92 (0.85–0.99)					
			2	0.83 (0.77–0.88)					
			≥3	0.71 (0.66–0.77)					
			(Database/Cancer Registry)						
La Vecchia, 1984, Italy, HC-CS	283/566 (33–74y)		Nulliparous	1.0 (ref.)	age				
			Parous	0.85 (0.69–1.05)					
			1	0.77 (0.58–1.01)					
			≥2	0.89 (0.72–1.11)					
			(Trained interviewer/histology confirmation)						
Salmi, 1979, Finland, PC-CS	282/282 (31–82y)		Nulliparous	1.0 (ref.)	Age, weight, social class				
			Parous	0.95 (0.79–1.15)					
			1–2	0.89 (0.72–1.10)					
			3–4	1.06 (0.84–1.32)					
			≥5	1.02 (0.72–1.44)					
			(Trained interviewer/histology confirmation)						
Asakura, 2009, Japan, PC-CS	191/419 (NA)		Nulliparous	1.0 (ref.)	Age, area, BMI				
			Parous	0.40 (0.26–0.61)					
			1	0.40 (0.22–0.74)					
			2	0.39 (0.25–0.61)					
			≥3	0.44 (0.24–0.79)					
			(questionnaire/histology confirmation)						
Hao, 2009, China, PC-CS	421/1263 (22–84y)		Nulliparous	1.0 (ref.)	Age, area				
			Parous	0.223 (0.115–0.435)					
			(questionnaire/cancer registry)						

BMI: body mass index; CI: confidence interval; CS: cohort study; HC-CS: hospital-based case-control study; NA: not available; NC-CS: nested case-control study; OR: odds ratio; PC-CS: population-based case-control study; ref.: reference; RR: relative risk.

**Table 2:** Quality Assessment of Reviewed Case-Control Studies.

**Study**	**Case defined with independent validation**	**Representativeness of the cases**	**Selection of controls from community**	**Statement that controls have no history of outcome**	**Cases and controls matched and/or adjusted by factors**	**Ascertain exposure by blinded structured interview**	**Same method of ascertainment for cases and controls**	**Same response rate for both groups**	**Overall Score**
Parslov, 2000	**1**	**1**	**1**	**1**	**2**	**0**	**1**	**1**	**8**
Salazar-Martinez, 1999	**1**	**1**	**0**	**0**	**2**	**1**	**1**	**1**	**7**
Parazzini, 1998	**1**	**1**	**0**	**0**	**2**	**1**	**1**	**1**	**7**
Kalandidi, 1996	**1**	**1**	**0**	**0**	**2**	**1**	**1**	**1**	**7**
Shu, 1993	**1**	**1**	**1**	**0**	**1**	**1**	**1**	**1**	**7**
Koumantaki, 1989	**1**	**1**	**0**	**0**	**0**	**1**	**1**	**1**	**5**
Kelsey, 1982	**1**	**1**	**0**	**1**	**2**	**1**	**1**	**1**	**8**
Baron, 1986	**1**	**1**	**0**	**1**	**0**	**1**	**1**	**1**	**6**
Castellsague, 1993	**1**	**1**	**1**	**0**	**2**	**1**	**1**	**1**	**8**
Dahlgren, 1991	**1**	**1**	**1**	**0**	**0**	**1**	**1**	**1**	**6**
Damon, 1960	**1**	**1**	**0**	**1**	**0**	**1**	**1**	**1**	**6**
Elwood, 1977	**1**	**1**	**1**	**0**	**1**	**0**	**1**	**1**	**6**
Fox, 1970	**1**	**1**	**1**	**0**	**1**	**1**	**1**	**1**	**7**
Garnet, 1958	**1**	**1**	**0**	**1**	**0**	**0**	**1**	**1**	**5**
Henderson, 1983	**1**	**1**	**1**	**0**	**1**	**1**	**1**	**1**	**7**
Hirose, 1996	**1**	**1**	**0**	**1**	**2**	**0**	**1**	**1**	**7**
Hosono, 2011	**1**	**1**	**0**	**1**	**2**	**0**	**1**	**1**	**7**
Jaakkola, 2011	**0**	**1**	**1**	**1**	**1**	**1**	**1**	**1**	**7**
Kakuta, 2009	**1**	**1**	**0**	**1**	**2**	**0**	**1**	**1**	**7**
Lawrence, 1989	**1**	**1**	**1**	**0**	**2**	**1**	**1**	**1**	**8**
Lesko, 1991	**1**	**1**	**0**	**1**	**2**	**1**	**1**	**1**	**8**
Levi, 1991	**1**	**1**	**0**	**1**	**0**	**1**	**1**	**1**	**6**
Littman, 2001	**1**	**1**	**1**	**1**	**2**	**1**	**1**	**1**	**9**
Macdonald, 1977	**1**	**1**	**1**	**1**	**1**	**1**	**1**	**1**	**8**
Newcomer, 2001	**1**	**1**	**1**	**1**	**1**	**1**	**1**	**1**	**8**
Pettersson, 1986	**1**	**1**	**1**	**1**	**2**	**1**	**1**	**1**	**9**
Spengler, 1981	**1**	**1**	**1**	**1**	**1**	**1**	**1**	**1**	**8**
Wynder, 1966	**1**	**1**	**0**	**1**	**0**	**1**	**1**	**1**	**6**
Wang, 1990	**1**	**1**	**0**	**1**	**2**	**1**	**1**	**1**	**8**
Hachisuga, 1998	**1**	**1**	**0**	**1**	**2**	**1**	**1**	**1**	**8**
Brons, 2015	**0**	**1**	**1**	**0**	**1**	**1**	**1**	**1**	**6**
La Vecchia, 1984	**1**	**1**	**0**	**1**	**1**	**1**	**1**	**1**	**7**
Salmi, 1979	**1**	**1**	**1**	**0**	**2**	**1**	**1**	**1**	**8**
Asakura, 2009	**1**	**1**	**1**	**1**	**2**	**0**	**1**	**0**	**7**
Hao, 2009	**0**	**1**	**1**	**1**	**2**	**0**	**1**	**1**	**7**

1 means study adequately fulfilled a quality criterion (2 for case-control fully matched and adjusted), 0 means it did not. Quality scale does not imply that items are of equal relevant importance.

**Table 3:** Quality Assessment of Reviewed Cohort Studies.

**Study**	**Exposed cohort represents average in community**	**Selection of the non-exposed cohort from same community**	**Ascertain exposure through records or structured interviews**	**Demonstrate that outcome not present at study start**	**Exposed and non-exposed matched and/or adjusted by factors**	**Ascertain outcome via independent blind assessment or record linkage**	**Follow-up long enough for outcome to occur**	**Loss to follow-up<20%**	**Overall Score**
Dossus, 2010	**1**	**1**	**0**	**1**	**2**	**1**	**1**	**1**	**8**
Wernli, 2006	**1**	**1**	**1**	**0**	**1**	**1**	**1**	**1**	**7**
Hinkula, 2002	**1**	**1**	**1**	**0**	**2**	**1**	**1**	**1**	**8**
Terry, 1999	**1**	**1**	**0**	**1**	**2**	**1**	**1**	**1**	**8**
Albrektsen, 1995	**1**	**1**	**1**	**0**	**2**	**1**	**1**	**1**	**8**
Kvale, 1988	**1**	**1**	**1**	**0**	**2**	**1**	**1**	**1**	**8**
PLCO, US	**1**	**1**	**0**	**1**	**2**	**1**	**1**	**1**	**8**
USRT, US	**0**	**1**	**0**	**1**	**2**	**1**	**1**	**1**	**7**
de Warrd, 1996	**1**	**1**	**0**	**0**	**0**	**1**	**1**	**1**	**5**
Bevier, 2011	**1**	**1**	**1**	**0**	**2**	**1**	**1**	**1**	**8**

1 means study adequately fulfilled a quality criterion, 0 means it did not. Quality scale does not imply that items are of equal relevant importance.

**Table 4:** Summary risk estimates of the association between parity and endometrial cancer risk (parous versus nulliparous).

	**No of reports**	**RR (95% CI)**	**I^2^ (%)**	**P for heterogeneity**
**Overall**	42	0.69 (0.65–0.74)	76.9	<0.001
Subgroup analysis				
Study design				
Prospective	7	0.66 (0.60–0.74)	0.0	0.790
Case–control	34	0.69 (0.64–0.74)	79.7	<0.001
Location				
Europe	15	0.76 (0.70–0.82)	67.9	<0.001
America	17	0.71 (0.64–0.78)	66.5	<0.001
Asia	9	0.53 (0.44–0.63)	58.6	0.013
International	1	0.73 (0.71–0.76)	—	—
Number of cases				
<200	19	0.68 (0.60–0.76)	57.1	0.001
≥200	23	0.70 (0.65–0.75)	83.3	<0.001
Study publication time				
Earlier than 1992	19	0.74 (0.68–0.82)	63.0	<0.001
1992–	23	0.66 (0.61–0.71)	82.8	<0.001
Estimate adjustment				
Yes	33	0.68 (0.63–0.73)	79.4	<0.001
No	9	0.72 (0.65–0.81)	52.1	0.033
Estimate adjusted for age				
Yes	32	0.68 (0.63–0.73)	80.1	<0.001
No	10	0.73 (0.66–0.81)	47.3	0.048
Estimate adjusted for BMI				
Yes	10	0.63 (0.51–0.77)	85.7	<0.001
No	32	0.71 (0.67–0.75)	72.7	<0.001
Estimate adjusted for smoking				
Yes	9	0.72 (0.61–0.85)	75.5	<0.001
No	33	0.68 (0.64–0.73)	77.8	<0.001
Estimate adjusted for age, BMI and smoking				
Yes	8	0.71 (0.59–0.85)	78.5	<0.001
No	34	0.69 (0.64–0.73)	77.1	<0.001
Sources of controls				
Population based	18	0.66 (0.60–0.73)	82.9	<0.001
Hospital based	16	0.72 (0.63–0.83)	76.3	<0.001
Study quality				
high	31	0.67 (0.62–0.73)	79.5	<0.001
low	10	0.72 (0.63–0.81)	65.7	0.002

**Table 5:** Summary risk estimates of the association between parity and endometrial cancer risk (parity number of 1 versus nulliparous).

	**No of reports**	**RR (95% CI)**	**I^2^ (%)**	**P for heterogeneity**
Parity number of 1 vs. nulliparous	19	0.73 (0.64–0.84)	88.3	<0.001
Subgroup analysis				
Study design				
Prospective	4	0.54 (0.40–0.72)	74.7	0.008
Case-control	14	0.83 (0.76–0.91)	35.2	0.093
Location				
Europe	9	0.70 (0.53–0.91)	92.8	<0.001
America	5	0.85 (0.77–0.93)	0.0	0.494
Asia	4	0.43 (0.29–0.63)	6.1	0.362
International	1	0.88 (0.84–0.92)	—	—
Number of cases				
<200	6	0.79 (0.64–0.97)	41.4	0.129
≥200	13	0.71 (0.60–0.84)	91.7	<0.001
Study publication time				
Earlier than 1992	7	0.81 (0.74–0.89)	0.0	0.782
1992–	12	0.66 (0.54–0.81)	92.7	<0.001
Estimate adjustment				
Yes	17	0.69 (0.58–0.82)	87.6	<0.001
No	2	0.89 (0.82–0.96)	5.7	0.303
Estimate adjusted for age				
Yes	17	0.69 (0.58–0.82)	87.6	<0.001
No	2	0.89 (0.82–0.96)	5.7	0.303
Estimate adjusted for BMI				
Yes	4	0.66 (0.43–1.01)	56.0	0.078
No	15	0.74 (0.63–0.86)	90.4	<0.001
Estimate adjusted for smoking				
Yes	3	0.86 (0.69–1.06)	0.0	0.496
No	16	0.72 (0.62–0.84)	90.1	<0.001
Estimate adjusted for age, BMI and smoking				
Yes	3	0.86 (0.69–1.06)	0.0	0.496
No	16	0.72 (0.62–0.84)	90.1	<0.001
Study quality				
high	15	0.67 (0.55–0.80)	83.2	<0.001
low	3	0.92 (0.85–0.99)	0.0	0.424

**Table 6:** Summary risk estimates of the association between parity and endometrial cancer risk (parity number of 2 versus nulliparous).

	**No of reports**	**RR (95% CI)**	**I^2^ (%)**	**P for heterogeneity**
Parity number of 2 vs. nulliparous	13	0.62 (0.53–0.74)	92.1	<0.001
Subgroup analysis				
Study design				
Prospective	2	0.54 (0.31–0.93)	92.7	<0.001
Case-control	10	0.63 (0.53–0.76)	68.8	0.001
Location				
Europe	7	0.61 (0.43–0.86)	95.3	<0.001
America	3	0.66 (0.54–0.80)	0.0	0.835
Asia	2	0.49 (0.31–0.78)	52.7	0.146
International	1	0.78 (0.75–0.81)	—	—
Number of cases				
<200	5	0.60 (0. 48–0.74)	16.2	0.312
≥200	8	0.64 (0.52–0.78)	95.1	<0.001
Study publication time				
Earlier than 1992	5	0.68 (0.58–0.79)	0.0	0.957
1992-	8	0.59 (0.47–0.74)	95.4	<0.001
Estimate adjustment				
Yes	11	0.59 (0.46–0.77)	92.5	<0.001
No	2	0.78 (0.75–0.81)	0.0	0.417
Estimate adjusted for age				
Yes	11	0.59 (0.46–0.77)	92.5	<0.001
No	2	0.78 (0.75–0.81)	0.0	0.417
Estimate adjusted for BMI				
Yes	4	0.50 (0.29–0.85)	79.5	0.002
No	9	0.66 (0.54–0.79)	94.0	<0.001
Estimate adjusted for smoking				
Yes	3	0.55 (0.27–1.09)	80.1	0.007
No	10	0.63 (0.53–0.76)	93.7	<0.001
Estimate adjusted for age, BMI and smoking				
Yes	3	0.55 (0.27–1.09)	80.1	0.007
No	10	0.63 (0.53–0.76)	93.7	<0.001
Study quality				
high	9	0.56 (0.44–0.73)	82.1	<0.001
low	3	0.74 (0.59–0.92)	52.1	0.124
