# Supplementary material for: Parity and endometrial cancer risk: a meta-analysis of epidemiological studies
Source: Sci Rep. 2015 Sep 16;5:14243. doi: 10.1038/srep14243 (PMC4642705; doi:10.1038/srep14243)
Supplement: Supplementary Information [file srep14243-s2.doc]

Subject Area: Public Health

**Parity and endometrial cancer risk: a meta-analysis of epidemiological studies**

Qi-Jun Wu^1#^, Yuan-Yuan Li^2#^, Chao Tu^3^, Jingjing Zhu^4,5^, Ke-Qing Qian^3^, Tong-Bao Feng^3^, Changwei Li^6^, Lang Wu^7,5*^, Xiao-Xin Ma^8*^

1 Department of Clinical Epidemiology, Shengjing Hospital of China Medical University, Shenyang 110004, Liaoning, China;

2 Department of Hematology, the Affiliated Hospital of Xuzhou Medical College, Xuzhou, Jiangsu, 221000, China;

3 Oncology Institute, the Affiliated Hospital of Nanjing Medical University, Changzhou No.2 People’s Hospital, Changzhou, Jiangsu,213003, China;

4 Program of Quantitative Methods in Education, University of Minnesota, Minneapolis, Minnesota, 55455, USA;

5 Division of Epidemiology, Department of Medicine, Vanderbilt Epidemiology Center, Vanderbilt University School of Medicine, Nashville, TN 37203, USA;

6 Department of Epidemiology, Tulane University School of Public Health and Tropical Medicine, New Orleans, Louisiana, 70112, USA;

7 Center for Clinical and Translational Science, Mayo Clinic, Rochester, Minnesota, 55905, USA

8 Department of Obstetrics and Gynecology, Shengjing Hospital of China Medical University, Shenyang 110004, Liaoning, China

# QJW and YYL contribute equally to this work.

**Corresponding Author**s: Xiao-Xin Ma, Department of Obstetrics and Gynecology, Shengjing Hospital of China Medical University, No. 36, San Hao Street, Shenyang 110004, Liaoning, China. Email: [Xiao_XM_sjhospital@163.com](mailto:Xiao_XM_sjhospital@163.com). Tel: 86-24-96615-42211; or Lang Wu, Center for Clinical and Translational Science, Mayo Clinic, 200 First Street SW, Rochester, MN 55905. Email: langlangcc@gmail.com. Tel: 1-507-293-1756.

**Running head**

Parity and risk of endometrial cancer

**Supplementary Figures**

Supplementary Figure 1. Studies contributing to heterogeneity in pooling analysis of the association between parity and endometrial cancer risk (parous versus nulliparous)


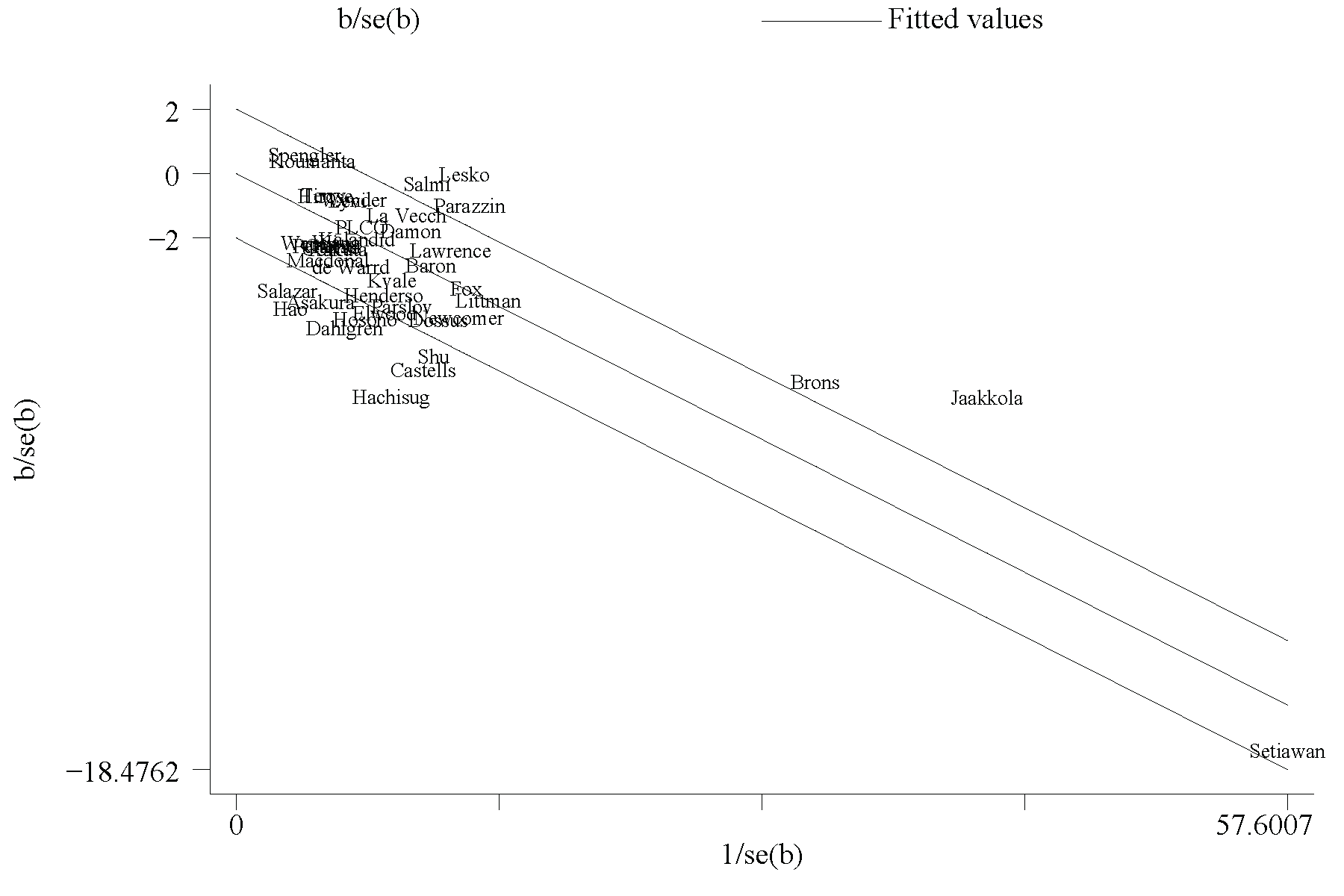


Supplementary Figure 2. Studies contributing to heterogeneity in pooling analysis of the association between parity and endometrial cancer risk (parity number of 1 versus nulliparous)


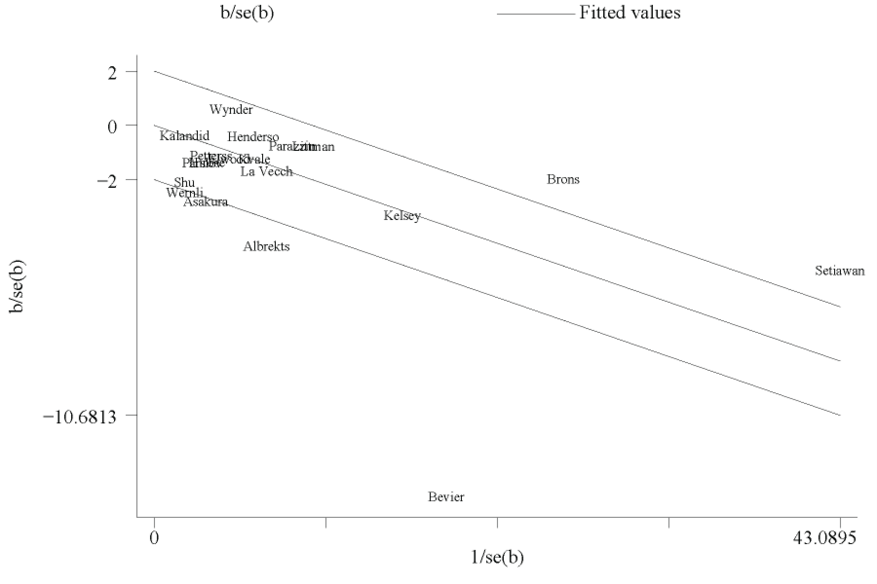


Supplementary Figure 3. Studies contributing to heterogeneity in pooling analysis of the association between parity and endometrial cancer risk (parity number of 2 versus nulliparous)


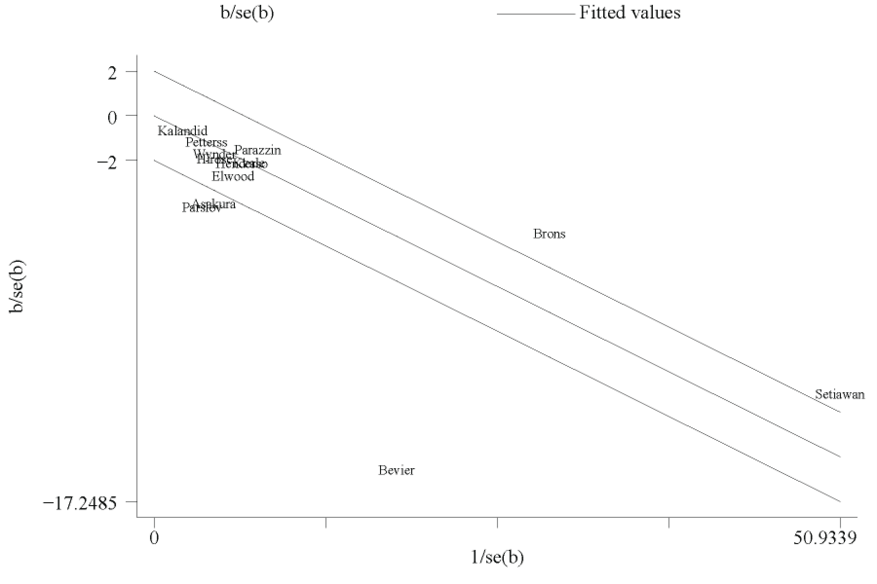


**Included studies in the current meta-analysis:**
